# Comparative effects of desflurane and sevoflurane on intraoperative peripheral perfusion index: a retrospective, propensity score matched, cohort study

**DOI:** 10.1038/s41598-022-27253-0

**Published:** 2023-03-06

**Authors:** Chahyun Oh, Seounghun Lee, Byong-Sop Song, Sanghun Kwon, Yoon-Hee Kim, Seok-Hwa Yoon, Yong Sup Shin, Youngkwon Ko, Chaeseong Lim, Boohwi Hong

**Affiliations:** 1 Department of Anesthesiology and Pain Medicine, Chungnam National University Hospital, 282 Munhwa-Ro, Jung-Gu, Daejeon, 35015 Korea; 2 Department of Anesthesiology and Pain Medicine, College of Medicine, Chungnam National University, Daejeon, South Korea; 3 Department of Anesthesiology and Pain Medicine, Chungnam National University Sejong Hospital, Sejong, Republic of Korea; 4 Core Laboratory of Translational Research, Biomedical Convergence Research Center, Chungnam National University Hospital, Daejeon, South Korea; 5 Big Data Center, Biomedical Research Institute, Chungnam National University Hospital, Daejeon, South Korea

**Keywords:** Cardiology, Health care, Medical research

## Abstract

Desflurane is known to have a larger vasodilatory effect than that of sevoflurane. However, its generalizability and effect size in actual clinical practice are yet to be proven. Patients aged ≥ 18 years who underwent noncardiac surgery under general anesthesia using inhalation anesthetics (desflurane or sevoflurane) were matched 1:1 by propensity score. The mean intraoperative perfusion index (PI) of each patient were compared between the two groups. Propensity score matching of 1680 patients in the study cohort identified 230 pairs of patients. PI was significantly higher in the desflurane group (median of paired difference, 0.45; 95% CI 0.16 to 0.74, p = 0.002). PI durations below 1.0 and 1.5 were significantly longer in the sevoflurane group. Mean arterial pressure (MAP) and durations of low MAP did not differ significantly between the two groups. Generalized linear mixed models revealed that the use of sevoflurane, mean MAP, mean heart rate, age, and duration of anesthesia had significant negative effects (lower PI), whereas mean age-adjusted minimum alveolar concentration of inhalation agent had a positive effect on PI (higher value). Intraoperative PI was significantly higher in patients administered desflurane than sevoflurane. However, the impact of the choice between desflurane and sevoflurane on intraoperative PI in this clinical setting was minimal.

## Introduction

Vascular tone is an essential factor for understanding organ perfusion^1^ and hemodynamic assessments^2,3^. Because organ perfusion does not depend solely on blood pressure, it is crucial to consider other parameters, such as vascular tone and regional perfusion^4^. Peripheral vascular tone and perfusion may be assessed by measuring the peripheral perfusion index (PI; or as PPI in some literature), a parameter derived from the pulse oximetry signal^4–6^. It represents the ratio of pulsatile to non-pulsatile components of the signal.

Inhalation anesthetics have vasodilatory effects^7,8^ and can improve microcirculation^9^. A recent randomized trial^6^ found that desflurane exerts a more potent vasodilatory effect than sevoflurane, as shown by higher PI and lower blood pressure. Although these findings were supported by the careful control of many possible confounders, such as comorbidities and stimulation intensity, the findings of that study may have limited generalizability to real-world clinical practice, in which such confounders cannot be controlled. Therefore, the present study retrospectively compared intraoperative PI in patients administered desflurane and sevoflurane as inhalation anesthetics.

## Materials and methods

The study was conducted in accordance with the principles of the Declaration of Helsinki and the study protocol was approved by the Institutional Review Board of Chungnam National University Hospital (CNUH 2021-11-008) on 12 November 2021 with waiver for informed consent and registered at the Clinical Research Information Service, a clinical trial registry in South Korea (KCT0006766).

### Study design

This retrospective cohort study included patients aged ≥ 18 years who underwent noncardiac surgery, including general, gynecological, otolaryngological, plastic, and urological surgery, under general anesthesia using inhalation anesthetics (desflurane or sevoflurane) and PI monitoring from February to August 2021 in a university hospital. Patients were excluded if clinical or vital data were missing, their vital records had interruptions, or if information was lacking regarding the inhalation agent, PI, or blood pressure (noninvasive or invasive arterial pressure). To account for the differences in their clinical characteristics, patients administered desflurane and sevoflurane were matched 1:1 by propensity score. This manuscript adheres to the applicable STROBE (Strengthening the Reporting of Observational Studies in Epidemiology) guidelines^10^.

### Data acquisition

All vital data were obtained from the prospective registry of the vital signs for surgical patients at Chungnam National University Hospital (CNUH IRB 2019-08-039), which uses a free data collection program (Vital recorder^11^ version 1.8, accessed at https://vitaldb.net, Seoul, Republic of Korea).

Other data collected from patient medical records included age, sex, body mass index (BMI), comorbidities (hypertension, diabetes, coronary artery disease, liver cirrhosis, chronic obstructive pulmonary disease, chronic renal impairment), Charlson comorbidity index, American Society of Anesthesiologists (ASA) physical status, type of surgery (general, gynecological, otolaryngological, plastic, or urological), emergency surgery, duration of anesthesia, intraoperative infusion of vasopressor (norepinephrine), intraoperative transfusion (red blood cells or fresh frozen plasma), intraoperative fluid input, and intraoperative opioid dose (remifentanil, μg kg^–1^ min^–1^).

Intraoperative PI and heart rate (HR) were monitored continuously using a disposable oximeter sensor (Nellcor™ Neonatal-Adult SpO_2_ sensor, Covidien, Mansfield, MA, USA) and a patient monitor (Intellivue MX700 or MX800 [Philips, Boeblingen, Germany]) and recorded at a frequency of 1 Hz. Oximeter sensor was routinely attached to the index or third finger of the patient unless contraindicated or inaccessible. Blood pressure was measured continuously with an arterial catheter or intermittently at 5-min intervals using a noninvasive blood pressure cuff and recorded at a frequency of 1 Hz. Intra-arterial pressure was primarily used for analysis, if available. Data regarding inhalation anesthetics (agent type, end-tidal concentration [%]) were obtained from the anesthesia machines and recorded at a frequency of 0.2–0.25 Hz. All vital signs and records of inhalation agents were extracted as 10 s interval mean values. These data were filtered for errors in blood pressure, so that the mean arterial pressure (MAP) was > 20 mmHg and < 150 mmHg. To include periods only with proper administration of inhalation anesthetics in the analysis, a cut-off value of end-tidal concentration (the 25th percentile of the intraoperative end-tidal concentration) of the inhalation agent was determined for the vital records of each individual and filtered accordingly. For example, if the median intraoperative end-tidal concentration of sevoflurane was 1.3 volume % (25th to 75th percentile, 1.2% to 1.4%), then the cut-off was set at 1.2 volume % and only periods with end-tidal sevoflurane concentrations above this value were included in the analysis. Mean of PI values acquired for three minutes immediately before the initiation of the administration of inhalation anesthetics was considered baseline.

### Outcome measures

The primary outcome was the mean intraoperative PI of each patient administered each inhalation agent. Other outcomes included the intraoperative durations at which the PI was below the thresholds of 0.5, 1, 1.5, and 2, mean HR, mean MAP, and the intraoperative durations at which MAP was below the thresholds of 50, 55, 60, 65, and 70 mmHg.

### Statistics

The sample size was based on data available during the study period. All statistical analyses were performed using R software version 4.0.3 (R Project for Statistical Computing, Vienna, Austria). Propensity scores were calculated using a logistic regression model, in which the type of inhalation agent was considered a dependent variable and the clinical characteristics of patients, including age, sex, BMI, Charlson comorbidity index, ASA physical status (1–2 or > 2), comorbidities, type of surgery, and intraoperative variables (duration of anesthesia, vasopressor infusion, transfusion, intravenous fluid intake, opioid, mean age-adjusted minimum alveolar concentration [MAC] of the inhalation agent^12^, and the administered dose of opioid), were considered explanatory variables. Patients were matched by 1:1 nearest neighbor matching using a caliper width of 0.09 (0.25 $\times$ the standard deviation [SD] of the propensity score) using the ‘MatchIt’ package^13^ in R software. The matching process included only those patients for whom we had complete records. The standardized mean difference was calculated to assess the balance between groups after matching, with a difference of < 0.1, indicating that the two groups were sufficiently balanced.

Continuous variables were reported as the mean ± SD or median (interquartile range [IQR]), depending on the results of the Shapiro–Wilk test. After the matching process, continuous variables were compared using paired Wilcoxon signed-rank tests^14^. Categorical variables were reported as numbers (%) and compared using chi-squared or Fisher’s exact tests. Statistical significance was set at a two-tailed p-value of < 0.05.

To identify factors affecting mean PI other than the inhalation agent, especially mean MAP and HR, a negative-binomial generalized linear mixed model was fitted. In that model, mean PI was considered a dependent variable; mean MAP, mean HR, inhalation agent, and the variables involved in the matching process were considered independent variables, and the matching identifier was considered a random effect. Because the dependent variable in the model could be reported only as a non-negative integer, the mean PIs were transformed as rounded values, expressed as 100 $\times$ mean PI. The proper fitting of the model was assessed using the ‘Diagnostics for HierArchical Regression Models (DHARMa)’ package^15^.

### Sensitivity analysis

Because the results can be influenced by the selection of specific matched data sets, the primary outcomes were analyzed and model fitting was performed using two additional matched datasets.

## Results

**Figure 1:**
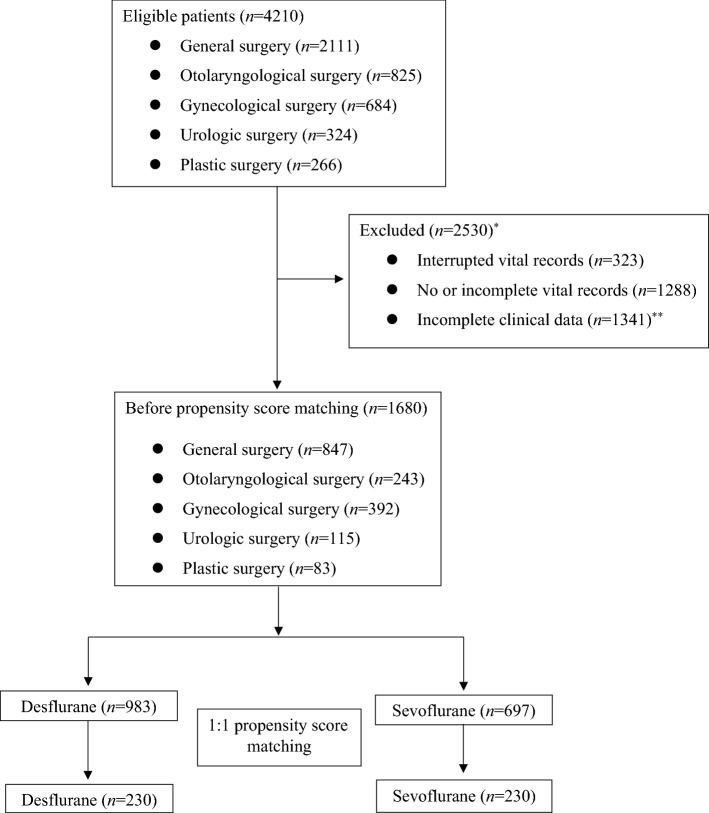
Patient flow chart. ^*^Includes duplicated reasons. ^**^Mainly due to intraoperative remifentanil dose (*n* = 1298).

A total of 4210 patients underwent noncardiac surgery under general anesthesia, including inhalation of desflurane or sevoflurane and PI monitoring from February to August 2021. Of these patients, 2530 were excluded due to insufficient data. The remaining 1680 patients were subjected to 1:1 propensity score matching, yielding a total of 230 pairs (Fig. 1). Clinical characteristics before and after matching are summarized in Table 1, with all clinical characteristics after matching adequately balanced.

**Table 1:** Clinical characteristics before and after propensity score matching.

	Before matching		SMD	After matching		SMD
	Desflurane	Sevoflurane		Desflurane	Sevoflurane	
	(*n* = 983)	(*n* = 697)		(*n* = 230)	(*n* = 230)	
Age (yr)	53.0 [42.0, 64.0]	55.0 [43.0, 67.0]	0.102	54.0 [44.0, 65.0]	52.5 [40.0, 67.0]	0.061
Sex (male)	376 (38.3)	284 (40.7)	0.051	82 (35.7)	80 (34.8)	0.018
BMI (kg m^–2^)	24.0 [21.9, 26.7]	24.0 [21.6, 26.7]	0.014	24.4 [22.3, 26.9]	24.0 [21.6, 27.0]	0.023
CCI*	5.0 [3.0, 7.0]	5.0 [3.0, 7.0]	0.058	5.0 [4.0, 7.0]	5.0 [3.0, 7.0]	0.028
ASA > 2	118 (12.0)	113 (16.2)	0.121	30 (13.0)	28 (12.2)	0.026
Duration of anesthesia (min)	1.6 [1.1, 2.3]	1.6 [1.2, 2.6]	0.099	1.6 [1.1, 2.3]	1.4 [1.0, 2.2]	0.018
Hypertension	274 (27.9)	204 (29.3)	0.031	72 (31.3)	66 (28.7)	0.057
Diabetes mellitus	136 (13.8)	93 (13.3)	0.014	32 (13.9)	35 (15.2)	0.037
Coronary artery disease	10 (1.0)	6 (0.9)	0.016	2 (0.9)	1 (0.4)	0.054
Chronic obstructive pulmonary disease	3 (0.3)	13 (1.9)	0.151	3 (1.3)	1 (0.4)	0.094
Liver cirrhosis	13 (1.3)	14 (2.0)	0.054	3 (1.3)	5 (2.2)	0.067
**Chronic renal dysfunction**						
On hemodialysis	7 (0.7)	13 (1.9)	0.106	4 (1.7)	4 (1.7)	< 0.001
No hemodialysis	15 (1.5)	13 (1.9)		4 (1.7)	4 (1.7)	
Emergency	37 (3.8)	100 (14.3)	0.375	19 (8.3)	14 (6.1)	0.084
**Type of surgery**						
Otolaryngology	203 (20.7)	40 (5.7)	0.493	20 (8.7)	17 (7.4)	0.081
General	451 (45.9)	396 (56.8)		129 (56.1)	125 (54.3)	
Gynecology	235 (23.9)	157 (22.5)		53 (23.0)	55 (23.9)	
Plastic	31 (3.2)	52 (7.5)		11 (4.8)	13 (5.7)	
Urology	63 (6.4)	52 (7.5)		17 (7.4)	20 (8.7)	
Vasopressor infusion	20 (2.0)	29 (4.2)	0.123	9 (3.9)	6 (2.6)	0.073
Transfusion	21 (2.1)	30 (4.3)	0.123	4 (1.7)	5 (2.2)	0.031
Intraoperative fluid intake (L)	0.5 [0.4, 0.8]	0.6 [0.4, 0.9]	0.117	0.5 [0.4, 0.8]	0.6 [0.4, 0.8]	0.008
Intraoperative remifentanil (μg kg^–^1 min^–^1)	0.1 [0.1, 0.1]	0.1 [0.1, 0.1]	0.105	0.1 [0.1, 0.1]	0.1 [0.1, 0.1]	0.013
Mean MAC	0.7 [0.6, 0.7]	0.8 [0.8, 0.9]	1.567	0.7 [0.7, 0.8]	0.7 [0.7, 0.8]	0.018

*SMD* standardized mean difference, *BMI* body mass index, *CCI* Charlson comorbidity index, *ASA* American Society of Anesthesiologists physical status, *MAC* minimum alveolar concentration. *Before index date (day of surgery). Data are reported as median (interquartile range) or number (%).

**Figure 2:**
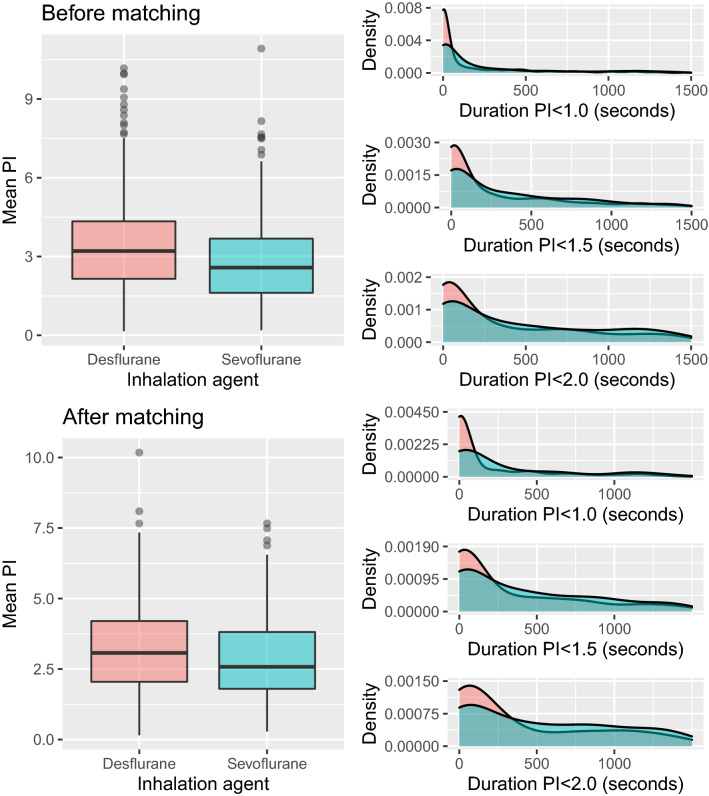
Box-and-whisker plot of mean perfusion index (PI) and kernel density plots of the duration of PIs below threshold stratified by the type of inhalation agent before (upper) and after (lower) the propensity score matching. Kernel density estimation is a non-parametric method of estimating the probability density function of a continuous random variable. The probability density function is used to specify the probability of the random variable falling within a particular range of the variable and the area under the entire curve is equal to one. Based on the presented kernel density plots, shorter durations of low PI values in patients administered desflurane than sevoflurane can be inferred.

The outcomes according to the administered inhalation agents are summarized in Table 2 and Fig. 2. There was no significant difference in the baseline PI. The mean PI values were significantly higher in the desflurane (median 3.1 [IQR 2.0, 4.2]) than in the sevoflurane (median 2.6 [IQR 1.8, 3.8]) group after matching (median paired difference, 0.45; 95% CI 0.16 to 0.74, p = 0.002). PI durations below 1.0 and 1.5 were significantly longer in the sevoflurane group than in the desflurane group. MAP and all defined durations of low MAP did not differ significantly between the two groups after matching.

**Table 2:** Outcomes according to inhalation agents before and after propensity score matching.

	Before matching		P*	After matching		p**
	Desflurane	Sevoflurane		Desflurane	Sevoflurane	
	(*n* = 983)	(*n* = 697)		(*n* = 230)	(*n* = 230)	
Baseline PI	3.2 [2.2, 4.3]	3.3 [2.1, 4.6]	0.757	3.2 [2.3, 4.3]	3.3 [1.9, 4.4]	0.333
Mean PI	3.2 [2.2, 4.3]	2.6 [1.6, 3.7]	< 0.001	3.1 [2.0, 4.2]	2.6 [1.8, 3.8]	0.002
PI < 0.5 (sec)	0.0 [0.0, 10.0]	0.0 [0.0, 70.0]	< 0.001	0.0 [0.0, 10.0]	0.0 [0.0, 60.0]	0.199
PI < 1.0 (sec)	20.0 [0.0, 420.0]	150.0 [0.0, 1170.0]	< 0.001	30.0 [0.0, 470.0]	170.0 [0.0, 1000.0]	0.019
PI < 1.5 (sec)	180.0 [0.0, 1065.0]	540.0 [50.0, 2140.0]	< 0.001	240.0 [10.0, 1170.0]	610.0 [80.0, 1600.0]	0.027
PI < 2.0 (sec)	500.0 [30.0, 1820.0]	1110.0 [230.0, 2920.0]	< 0.001	670.0 [70.0, 1830.0]	1015.0 [250.0, 2210.0]	0.092
Mean HR (BPM)	70.0 [63.0, 78.1]	70.3 [63.0, 79.7]	0.452	71.1 [64.3, 79.4]	69.4 [61.9, 77.2]	0.168
Mean MAP (mmHg)	79.8 [73.7, 86.9]	81.1 [75.9, 87.6]	< 0.001	80.2 [74.9, 88.5]	80.5 [74.6, 86.9]	0.681
MAP < 50 mmHg (sec)	0.0 [0.0, 0.0]	0.0 [0.0, 0.0]	0.775	0.0 [0.0, 0.0]	0.0 [0.0, 0.0]	0.681
MAP < 55 mmHg (sec)	0.0 [0.0, 0.0]	0.0 [0.0, 0.0]	0.614	0.0 [0.0, 0.0]	0.0 [0.0, 0.0]	0.374
MAP < 60 mmHg (sec)	0.0 [0.0, 140.0]	0.0 [0.0, 80.0]	0.061	0.0 [0.0, 130.0]	0.0 [0.0, 100.0]	0.341
MAP < 65 mmHg (sec)	90.0 [0.0, 580.0]	50.0 [0.0, 380.0]	0.022	5.0 [0.0, 440.0]	90.0 [0.0, 420.0]	0.495
MAP < 70 mmHg (sec)	470.0 [0.0, 1385.0]	330.0 [0.0, 1060.0]	0.001	395.0 [0.0, 1230.0]	425.0 [0.0, 1120.0]	0.366

*PI* perfusion index, *HR* heart rate, *BPM* beat per minute, *MAP* mean arterial pressure.

*Results of unpaired tests. **Results of paired tests. Continuous data are reported as median (interquartile range).

**Figure 3:**
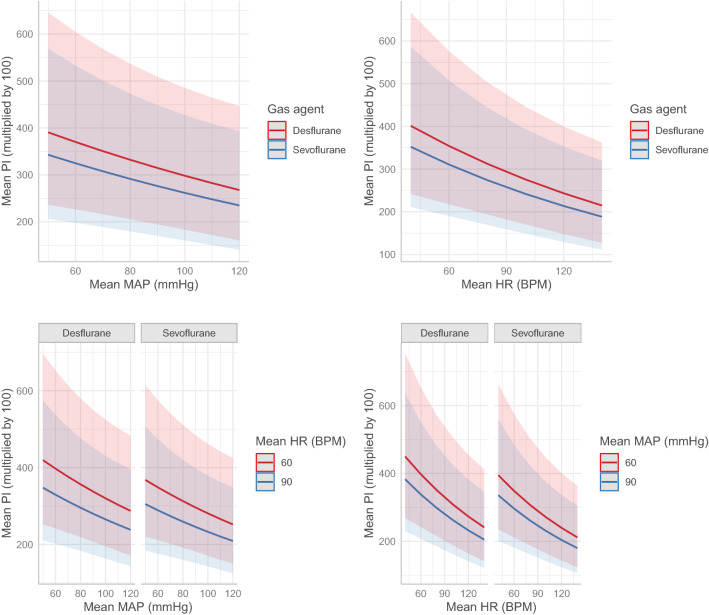
Effect of mean arterial pressure (MAP) and heart rate (HR) on perfusion index (PI) stratified by the type of inhalation agent. The plot presents the estimated marginal mean PI derived from the mixed-effects model. The shaded areas represent 95% confidence intervals of the estimated means. Note that the PI on the y-axis is multiplied by 100. *BPM* beat per minute.

The regression model showed that administration of sevoflurane, higher mean MAP, higher mean HR, older age, ASA > 2, longer duration of anesthesia, and certain type of surgery (general, gynecologic, and urologic surgery) had significant negative effects on mean PI (lower value), whereas chronic obstructive pulmonary disease and higher mean age-adjusted MAC of inhalation agent had significant positive effects on mean PI (higher value; Table 3). Specifically, the use of sevoflurane lowered the mean PI by 12.3%. In addition, a 10 mmHg increase in mean MAP and mean HR lowered mean PI by 5.3% and 6.1%, respectively. The relationship between mean MAP, mean heart rate, and mean PI stratified by the type of inhalation agent is shown in Fig. 3.

**Table 3:** Summary of the generalized linear mixed model.

Predictors	Estimate	Exp (estimate)*	95% CI	*P*
Inhalation agent (sevoflurane)	− 0.131	0.88	0.81–0.95	0.002
Mean MAP (10 mmHg)**	− 0.054	0.95	0.90–0.99	0.022
Mean HR (10 BPM)**	− 0.063	0.94	0.90–0.98	0.001
Age (yr)	− 0.005	1.00	0.99–1.00	0.019
Sex (male)	− 0.026	0.97	0.88–1.08	0.628
BMI (kg m^−2^)	− 0.006	0.99	0.98–1.01	0.371
CCI	− 0.002	1.00	0.97–1.02	0.874
ASA (> 2)	− 0.176	0.84	0.71–0.99	0.040
Duration of anesthesia (hr)	− 0.159	0.85	0.80–0.91	< 0.001
Hypertension	0.003	1.00	0.89–1.12	0.962
Diabetes mellitus	0.011	1.01	0.87–1.17	0.884
Coronary artery disease	0.139	1.15	0.71–1.86	0.573
Chronic obstructive pulmonary disease	0.426	1.53	1.01–2.31	0.043
Liver cirrhosis	− 0.016	0.98	0.70–1.38	0.928
**Chronic renal dysfunction**				
On hemodialysis	0.194	1.21	0.82–1.79	0.325
No hemodialysis	0.201	1.22	0.87–1.72	0.248
Emergency	− 0.096	0.91	0.76–1.09	0.300
Type of surgery***				
General	− 0.202	0.82	0.70–0.96	0.011
Gynecology	− 0.321	0.73	0.60–0.87	0.001
Plastic	0.001	1.00	0.79–1.26	0.995
Urology	− 0.262	0.77	0.62–0.95	0.016
Vasopressor infusion	− 0.192	0.83	0.61–1.12	0.223
Transfusion	− 0.448	0.64	0.40–1.03	0.064
Intraoperative fluid intake (mL)	0.099	1.10	0.98–1.24	0.093
Remifentanil (μg kg^−1^ min^−1^)	− 0.305	1.36	0.26–7.03	0.716
Mean MAC	0.990	2.69	1.45–4.99	0.002

*MAP* mean arterial pressure, *HR* heart rate, *BPM* beat per minute, *BMI* body mass index, *CCI* Charlson comorbidity index, *ASA* American Society of Anesthesiologists physical status, *MAC* minimum alveolar concentration.

*Exponential of the estimate. **Re-scaled using tenfold multiplication. ***Otolaryngological surgery was considered reference.

The results of the sensitivity analysis are shown in Supplementary Materials 1 and 2. The result of the primary outcome was consistent in the additional datasets. Administration of sevoflurane, mean MAP, mean HR, age, and duration of anesthesia had significant negative effects on mean PI, whereas mean age-adjusted MAC of inhalation agent had significant positive effect on mean PI regardless of the dataset used.

## Discussion

The present study was performed to assess generalizability of the findings from a previous clinical trial in a real-world data with propensity score matching. In line with the previous trial, the current study showed that PI was higher in patients receiving desflurane than in those receiving sevoflurane. In addition, the duration of low PI was longer in the sevoflurane group. However, the impact of the choice between desflurane and sevoflurane on intraoperative PI was minimal, median 0.45 and about 12% in the multivariable model, which has questionable clinical significance.

The previous experimental study revealed that the use of desflurane result in mean 3.3 difference of PI compared with the use of sevoflurane and significant difference in blood pressure. However, the amount of PI difference was much lesser and no significant difference in blood pressure were noted depending on the inhalation agent in the current study. During dynamic intraoperative period, many factors such as various surgical stimulation, opioid use, and fluid and/or vasopressor administrations coexist and can interfere with the impact of inhalation agent on PI. Moreover, mixed population with various comorbidities and surgical procedure can surely be important confounding factors. As aforementioned factors cannot be completely controlled in a real-world setting, the findings in the current study would be more pragmatic than the previous one^16,17^. In other words, choice or switching between the two inhalation agents for the purpose of manipulating intraoperative PI seems impractical.

This study has several strengths. First, we used real-world data consisting of high-resolution vital records. The second strength was the use of a propensity score matching process, which resulted in a proper balance between the two groups. This process involves the consideration of various confounders, not only baseline characteristics but also intraoperative variables, including the mean MAC of the inhalation agent, opioid dose, and fluid intake. Third, the regression analysis after matching provides “double robustness”, complementing the findings of propensity score matching^18^. Fourth, further robustness was provided by sensitivity analysis using different sets of propensity score-matched patients. Fifth, a retrospective design using a dataset from a different institution may alleviate performance bias and generalizability could have been assessed.

The present study assumed PI is an indicator of vasodilation. Although this assumption is supported by several previous studies^6,19–22^, other studies suggest that PI also depends on cardiac output or stroke volume^23–25^. Considering relevant evidences altogether, it is more reasonable to consider PI as a combinational parameter dependent on various local and systemic factors^26^. Following this logic, the impact of inhalation agent on PI is more likely to be complex rather than simply reflecting vasodilation.

Other factors found to be consistently associated with mean PI in this study included mean MAP, mean HR, age, duration of anesthesia, and age-adjusted MAC. According to a recent review article^26^, PI is mainly determined by stroke volume and vascular tone. Increase of vascular tone and decrease of stroke volume can lead to reduced PI, and vice versa. In this context, the negative coefficient of mean MAP in the models indicate high vascular tone manifested as low PI with a given stroke volume. The negative coefficients of mean HR in the models may reflect decreased stroke volume and/or increased vascular tone, which is commonly accompanied by volume deficit and resultant increase of HR. In addition, the coefficient of mean age-adjusted MAC reflects positive dose–response relationship between inhalation agent and PI. However, these inferences should be considered hypothesis generating and needs further studies. Especially, caution is needed as those relationships between MAP, HR, and PI were not evaluated on an individual patient level with serial measurements.

Recent evidence has suggested that clinicians should pay attention not only to blood pressure but also to perfusion during surgery^1,4,5,27^. Because organ perfusion is not solely dependent on blood pressure, but is also affected by regional vascular tone, the suggestion that greater vasodilation and lower blood pressure arising from the use of desflurane may result in potential clinical harm requires careful reconsideration^6^. Because vasodilation and low blood pressure may exert opposite effects on organ perfusion, the net effect cannot be estimated simply. For example, the negative effect of desflurane-induced low blood pressure on organ perfusion may be counteracted by desflurane-induced vasodilation. Future studies should analyze these complicated net effects.

This study had several limitations. First, despite the availability of high-resolution vital records, only descriptive statistics (i.e. mean PI and MAP), rather than the original values, were included in the final analysis, except for the durations of low PI and MAP. Second, a large number of patients could not be included in the analysis because of the lack of vital recordings or detailed clinical information (mostly intraoperative remifentanil doses which were manually recorded). Although selection bias due to this issue cannot be excluded, these omissions were more likely to be random (missing at random) rather than systematic. Third, more detailed information, such as bolus use of a vasopressor (e.g. phenylephrine) and the total dose of infused norepinephrine, was not included in the analysis due to unreliable quality. Instead, the use of norepinephrine infusion was included in the analysis as a surrogate marker of considerable requirement of vasopressor because it is commonly adopted after repeated doses of phenylephrine. Fifth, subtle discrepancies in intraoperative management may exist among clinicians, some of whom may prefer either of the inhalation agents. This might have affected the results of the current study.

In conclusion, despite the lack of different effect of inhalation agents on MAP, the mean PI was significantly higher in patients administered desflurane than sevoflurane. However, the impact of the choice between desflurane and sevoflurane on intraoperative PI in this clinical setting was minimal which has questionable clinical significance.

## Supplementary Information

Supplementary Information 1.

Supplementary Information 2.

## Data Availability

The data of the current study are available from the corresponding author on reasonable request.
