# Supplementary material for: Comparative effects of desflurane and sevoflurane on intraoperative peripheral perfusion index: a retrospective, propensity score matched, cohort study
Source: Sci Rep. 2023 Mar 6;13:2991. doi: 10.1038/s41598-022-27253-0 (PMC9988875; doi:10.1038/s41598-022-27253-0)
Supplement: Supplementary file 2 — Supplementary Information 2. [file 41598_2022_27253_MOESM2_ESM.docx]

**Supplementary material 2**

The summary results derived from the additional matched data set (2^nd^)

Clinical characteristics after the matching

|  | After matching | |  |
| --- | --- | --- | --- |
|  | Desflurane | Sevoflurane | SMD |
|  | (*n* =231) | (*n* =231) |  |
| Age (yr) | 53.0 [43.0, 65.0] | 51.0 [40.0, 66.0] | 0.055 |
| Sex (male) | 78 (33.8) | 78 (33.8) | <0.001 |
| BMI (kg m^–2^) | 24.2 [22.0, 27.0] | 24.0 [21.6, 27.0] | 0.016 |
| CCI^*^ | 5.0 [4.0, 7.0] | 5.0 [ 3.0, 7.0] | 0.020 |
| ASA>2 | 33 (14.3) | 27 (11.7) | 0.077 |
| Duration of anesthesia (min) | 1.6 [1.1, 2.3] | 1.4 [1.0, 2.2] | 0.003 |
| Hypertension | 65 (28.1) | 64 (27.7) | 0.010 |
| Diabetes mellitus | 30 (13.0) | 34 (14.7) | 0.050 |
| Coronary artery disease | 2 (0.9) | 1 (0.4) | 0.054 |
| Chronic obstructive pulmonary disease | 3 (1.3) | 1 (0.4) | 0.094 |
| Liver cirrhosis | 1 (0.4) | 4 (1.7) | 0.126 |
| Chronic renal dysfunction |  |  | 0.066 |
| On hemodialysis | 4 (1.7) | 4 (1.7) |  |
| No hemodialysis | 5 (2.2) | 3 (1.3) |  |
| Emergency | 18 (7.8) | 14 (6.1) | 0.068 |
| Type of surgery |  |  | 0.129 |
| Otolaryngology | 21 (9.1) | 17 (7.4) |  |
| General | 119 (51.5) | 124 (53.7) |  |
| Gynecology | 63 (27.3) | 57 (24.7) |  |
| Plastic | 14 (6.1) | 13 (5.6) |  |
| Urology | 14 (6.1) | 20 (8.7) |  |
| Vasopressor infusion | 7 (3.0) | 6 (2.6) | 0.026 |
| Transfusion | 6 (2.6) | 4 (1.7) | 0.060 |
| Intraoperative fluid intake (L) | 0.6 [0.4, 0.8] | 0.6 [0.4, 0.8] | 0.007 |
| Intraoperative remifentanil  (μg kg–1 min–1) | 0.1 [0.1, 0.1] | 0.1 [0.1, 0.1] | 0.048 |
| Mean MAC | 0.7 [ 0.7; 0.8] | 0.7 [ 0.7; 0.8] | 0.014 |

SMD, standardized mean difference; BMI, body mass index; CCI, Charlson comorbidity index; ASA, American Society of Anesthesiologists physical status; MAC, minimum alveolar concentration. ^*^Before index date (day of surgery). Data are reported as median (interquartile range) or number (%).


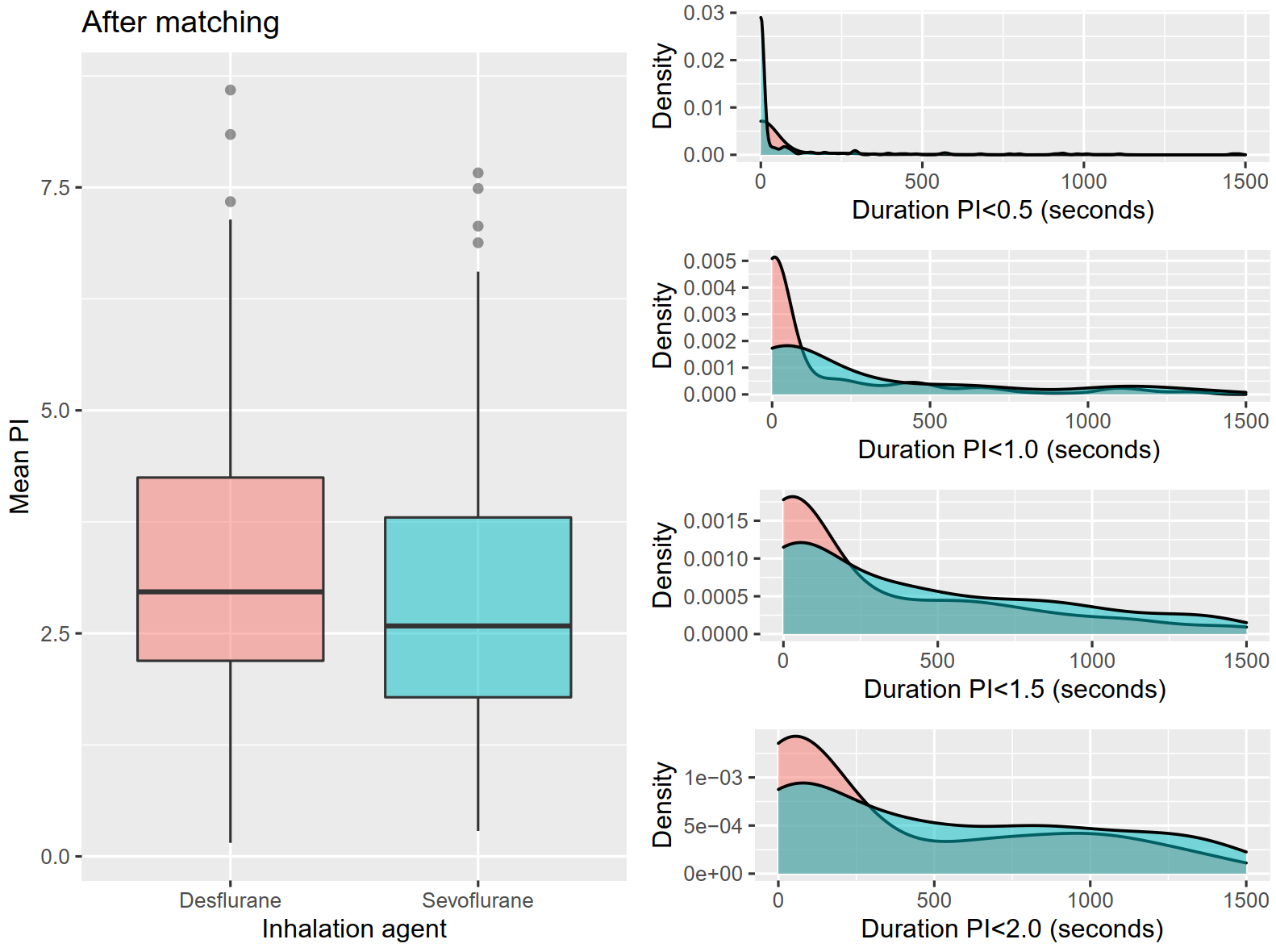


Box-and-whisker plot for mean perfusion index (PI) and kernel density plot for the duration of below threshold PIs stratified by the type of inhalation agent after propensity score matching.

|  | Desflurane | Sevoflurane | *P^*^* |
| --- | --- | --- | --- |
|  | (n=231) | (n=231) |  |
| Baseline PI | 3.3 [2.3, 4.3] | 3.2 [1.9, 4.4] | 0.386 |
| Mean PI | 3.0 [2.2, 4.2] | 2.6 [1.8, 3.8] | 0.005 |

^*^ Result of paired test. Data are given as median [IQR]

Summary of the generalized linear mixed model

| Predictors | Exp (estimate)^*^ | 95% CI | *P* |
| --- | --- | --- | --- |
| Inhalation agent (sevoflurane) | 0.85 | 0.79 – 0.92 | <0.001 |
| Mean MAP (10 mmHg)^**^ | 0.92 | 0.88 – 0.96 | 0.001 |
| Mean HR (10 BPM)^**^ | 0.95 | 0.92 – 0.99 | 0.012 |
| Age (yr) | 0.99 | 0.99 – 1.00 | 0.008 |
| Sex (male) | 0.95 | 0.85 – 1.05 | 0.322 |
| BMI (kg m^–2^) | 1 | 0.98 – 1.01 | 0.547 |
| CCI | 1 | 0.98 – 1.03 | 0.937 |
| ASA (>2) | 0.9 | 0.77 – 1.05 | 0.192 |
| Duration of anesthesia (hr) | 0.86 | 0.81 – 0.91 | <0.001 |
| Hypertension | 0.94 | 0.84 – 1.06 | 0.307 |
| Diabetes mellitus | 1.13 | 0.98 – 1.29 | 0.085 |
| Coronary artery disease | 1.21 | 0.75 – 1.94 | 0.441 |
| Chronic obstructive pulmonary disease | 1.48 | 0.99 – 2.21 | 0.054 |
| Liver cirrhosis | 1.11 | 0.75 – 1.65 | 0.599 |
| Chronic renal dysfunction |  |  |  |
| - On hemodialysis | 1.14 | 0.78 – 1.66 | 0.506 |
| - No hemodialysis | 1.3 | 0.95 – 1.79 | 0.096 |
| Emergency | 0.89 | 0.75 – 1.07 | 0.221 |
| Type of surgery^***^ |  |  |  |
| - General | 0.84 | 0.73 – 0.98 | 0.029 |
| - Gynecology | 0.75 | 0.63 – 0.89 | 0.001 |
| - Plastic | 1.11 | 0.90 – 1.37 | 0.351 |
| - Urology | 0.77 | 0.62 – 0.95 | 0.017 |
| Vasopressor infusion | 0.74 | 0.53 – 1.02 | 0.070 |
| Transfusion | 0.51 | 0.32 – 0.81 | 0.004 |
| Intraoperative fluid intake (mL) | 1.07 | 0.95 – 1.20 | 0.292 |
| Remifentanil (μg kg^–1^ min^–1^) | 1.15 | 0.24 – 5.54 | 0.859 |
| Mean MAC | 1.95 | 1.09 – 3.50 | 0.025 |

MAP, mean arterial pressure; HR, heart rate; BPM, beat per minute; BMI, body mass index; CCI, Charlson comorbidity index; ASA, American Society of Anesthesiologists physical status; MAC, minimum alveolar concentration ^*^Exponential of the estimate. ^**^Re-scaled using 10-fold multiplication. ^***^Otolaryngological surgery was considered reference.


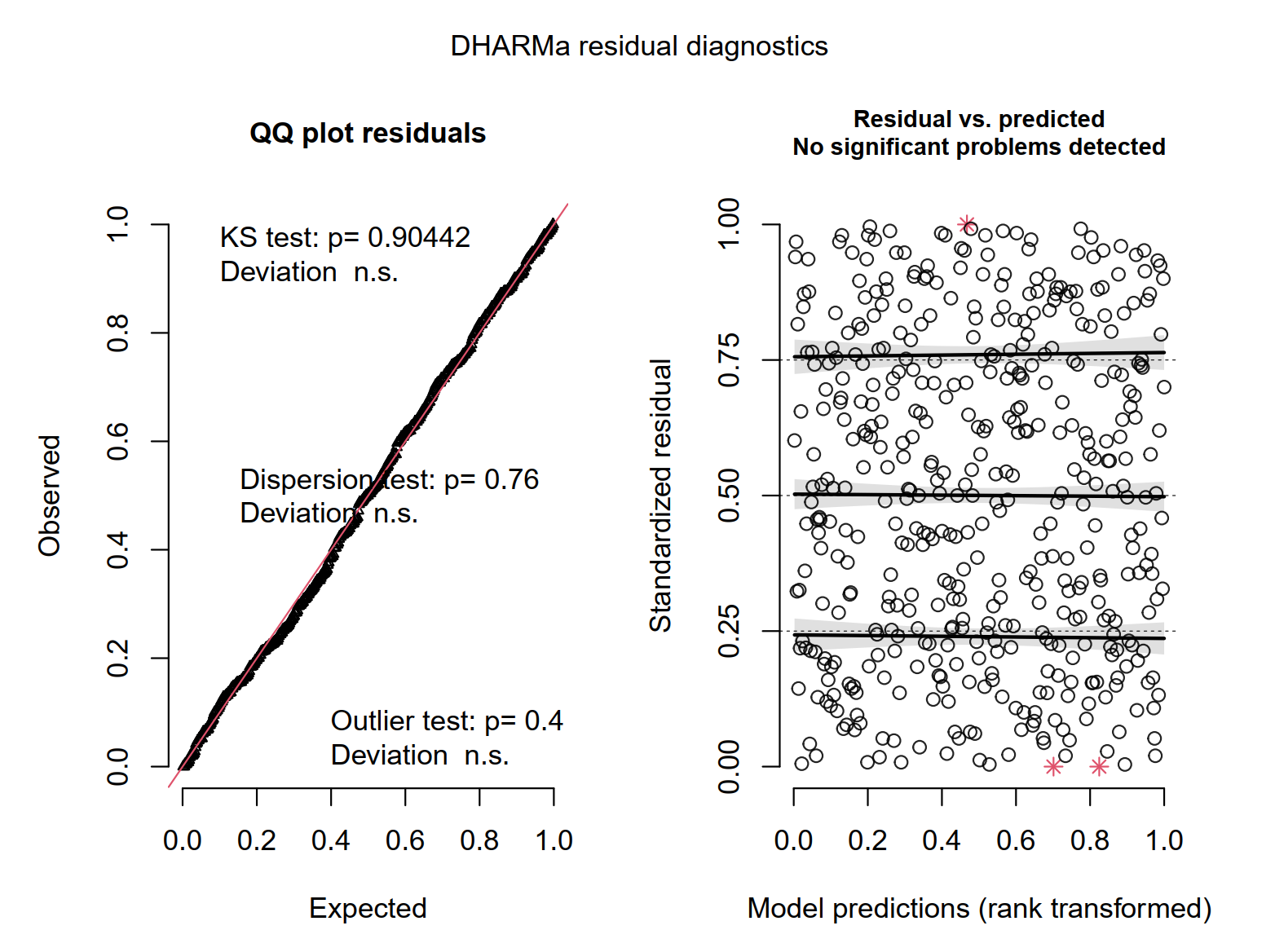


**Result of the model diagnostics using DHARMa package**
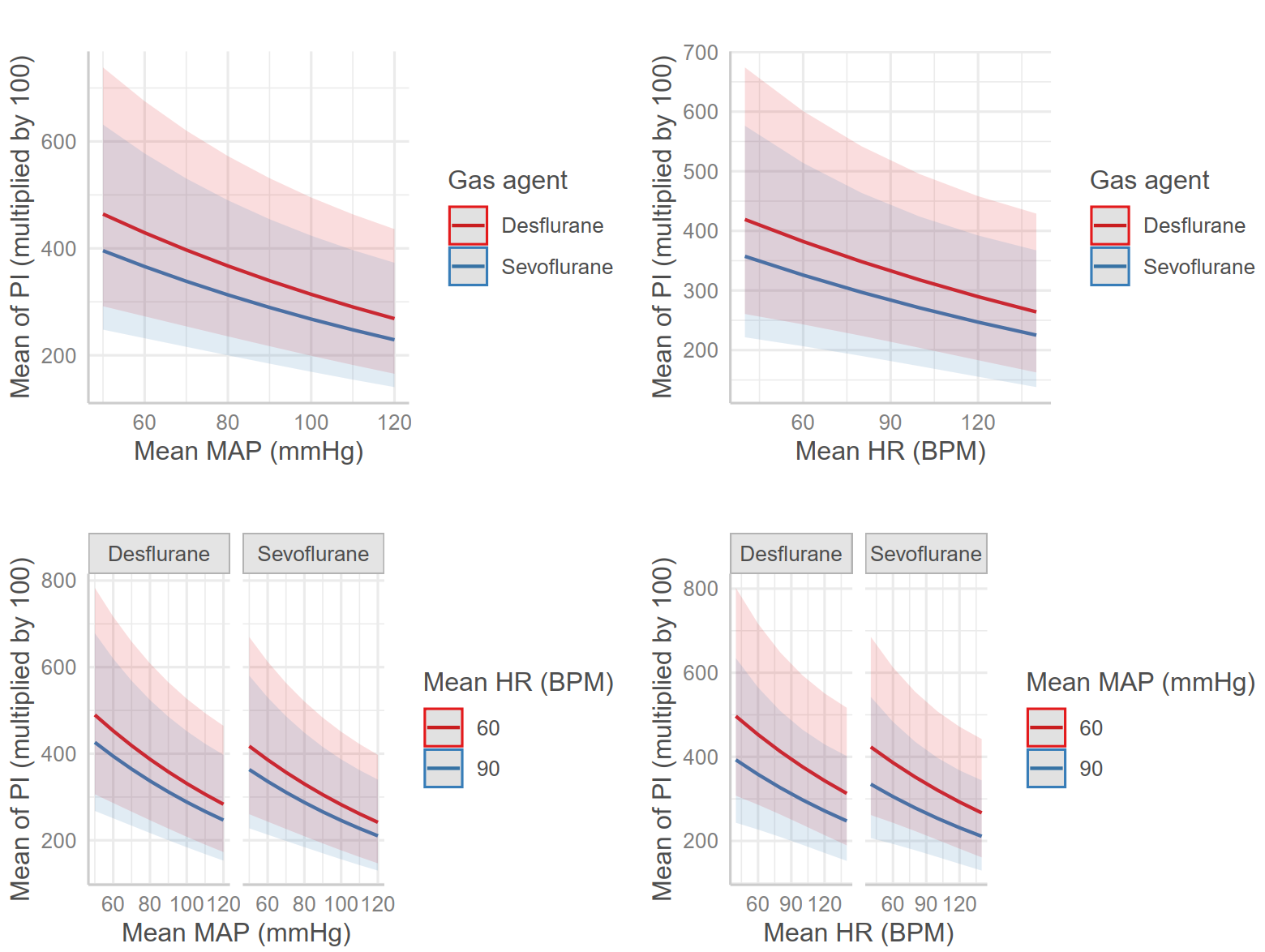


The relationship between mean MAP, mean heart rate, and mean PI stratified by the type of inhalation agent

The plot presents the estimated marginal mean of the mean PI derived from the mixed effect model. The shaded areas represent the 95% confidence interval of the estimated means. Note that the perfusion index on y-axis is multiplied by 100.
